# *Histoplasma capsulatum* Infection in an Allogeneic Hematopoietic Stem Cell Transplant Patient Receiving Voriconazole Prophylaxis

**DOI:** 10.1155/2020/8124137

**Published:** 2020-02-12

**Authors:** Vaibhav Agrawal, Bryan J. Brinda, Sherif S. Farag

**Affiliations:** ^1^Department of Medicine, Division of Hematology and Oncology, Indiana University School of Medicine, Indianapolis, IN, USA; ^2^Bone Marrow and Blood Stem Cell Transplantation Program, Indiana University, Indianapolis, IN, USA; ^3^Department of Pharmacy, Division of Hematology-Oncology, University of Kentucky, Lexington, KY, USA

## Abstract

*Histoplasma capsulatum* infection is a rare complication in the allogeneic stem cell transplant patients. Minimal guidance exists on how to appropriately manage histoplasmosis in these patients. We report a patient who developed *Histoplasma* pneumonia while receiving voriconazole prophylaxis at a therapeutic trough level. The patient experienced significant clinical improvement after initiation of itraconazole pharmacotherapy. We recommend a lower threshold for evaluation for histoplasmosis in allogeneic hematopoietic stem cell transplant recipients who live in endemic regions, regardless of their antifungal prophylactic regimen.

## 1. Introduction

Within the United States, most cases of *Histoplasma capsulatum* infection have been reported in the Ohio and Mississippi River valleys. While an otherwise asymptomatic *H. capsulatum* infection can occur in patients with competent immune systems, it is far more clinically concerning in those who are immunosuppressed [1]. Typical signs and symptoms of *H. capsulatum* infection include fever, chills, myalgias, dry cough, and chest discomfort. In immunocompromised patients, the disease may become disseminated and if untreated, is usually fatal with a reported mortality rate of 67% in allogeneic hematopoietic stem cell transplant recipients [2]. Itraconazole and amphotericin are the first-line therapies for treatment of histoplasmosis; however, other antifungal agents such as voriconazole and posaconazole are thought to be active and have been utilized as salvage therapy [3]. Here, we present a unique case of *H. capsulatum* infection in a matched-unrelated donor stem cell transplant patient who was receiving voriconazole prophylaxis.

## 2. Case Presentation

A 55-year-old male who was a long-term resident of Indiana with a history of allogenic peripheral stem cell transplantation presented to the bone marrow transplant clinic for routine follow-up. He initially was diagnosed with stage IVB diffuse large B-cell lymphoma in August 2009 and was treated with rituximab, cyclophosphamide, doxorubicin, vincristine, and prednisone (R-CHOP) and achieved complete remission. He relapsed in April 2010 and was treated with rituximab, etoposide, methylprednisolone, high-dose cytarabine, and cisplatin (R-ESHAP) and underwent an autologous peripheral stem cell transplant in July 2010 with the myeloablative preparative regimen of carmustine, etoposide, cytarabine, and melphalan (BEAM). In July 2015, he developed therapy-related myelodysplastic syndrome after his autologous transplant and underwent initial cytoreduction with 5-azacytidine followed by a matched-unrelated donor allogeneic peripheral blood stem cell transplantation in June 2016 at another institution. The patient developed acute graft-versus-host disease (GVHD) involving the skin and gut approximately one month after transplantation, which responded to corticosteroids as well as continuation of calcineurin inhibitor. He continued to be treated intermittently with courses of corticosteroids for presumed chronic GVHD of the liver until day 282 after transplant when he switched his care to our institution. At this point, he was found to have no evidence of active GVHD except for a mild elevation of alkaline phosphatase; corticosteroids were therefore tapered and discontinued on day 329 after transplant. He was also found to have anemia and thrombocytopenia, which was shown to be due to thrombotic microangiopathy, and calcineurin inhibitor therapy was also discontinued and the patient was placed on mycophenolate mofetil to prevent any exacerbations of chronic GVHD.

On day +365 after transplant, he presented to the clinic with a cough productive of whitish sputum, fatigue, weakness, and intermittent fevers with chills. A chest X-ray showed no radiographic abnormalities, and a respiratory viral panel was positive for parainfluenza virus type 3. Blood cultures were negative. No abnormalities were noted in his blood counts or complete metabolic panel. His anti-infectious regimen at the time included prophylaxis with atovaquone 1500 mg daily for prevention of pneumocystis pneumonia, valacyclovir 500 mg daily for prevention of zoster infections, and voriconazole 200 mg twice daily for prevention of fungal infections in the setting of his increased risk secondary to long-term steroid exposure. Initial recommendations were to proceed with supportive care therapeutic strategies for his parainfluenza virus type 3. He returned to the clinic two weeks later and while reporting general improvement in his respiratory symptoms, he continued to experience chills and sweating at night without any documented fever. No antibacterial therapy was prescribed at this time, and continued therapeutic intervention with supportive care was recommended. Five days later, he returned to the clinic with complaints of wheezing, green-yellow sputum production, low-grade fevers, continued fatigue, and nausea. Blood cultures, sputum cultures, and serum cytomegalovirus were all negative. He received nebulized albuterol and a prescription for clarithromycin. A computed tomography (CT) scan of the chest was performed that revealed diffuse peribronchial wall thickening with tree in bud nodularity in the lower lobes, two pulmonary nodules within the left apex each measuring 3 mm, and a 5 mm nodule within the right lower lobe. A repeat respiratory viral panel showed the persistence of both parainfluenza virus 3 and now newly detected respiratory syncytial virus A.

As the patient was not clinically improving and continued to report low-grade fever a week later, bronchoscopy was performed. Bronchoalveolar lavage (BAL) fluid showed pink and cloudy secretions with 6% monocytes, 93% granulocytes, and 1% eosinophils. Calcofluor-white and Gram stains were negative for *Aspergillus* and blastomycosis, as well as for *Histoplasma*. A positive antigen for *Histoplasma* was detected in the bronchoalveolar lavage sample using MiraVista diagnostic testing, but below the limit of quantification (reported as above the limit of detection but <0.4 ng/mL). A urine *Histoplasma* test was negative. At this time, a voriconazole serum trough level was 2.4 mcg/mL (goal 1–5.5 mcg/mL). He was also evaluated in a consultative visit by Infectious Disease to affirm this diagnosis of histoplasmosis. With concern for the patient's worsening clinical condition in his immunocompromised state and the lack of access to molecular testing at the time to support the result of antigen testing, recommendations were made to discontinue voriconazole and commence treatment with itraconazole 200 mg twice daily by mouth and mycophenolate mofetil was discontinued.

The patient returned to the clinic two weeks later and reported resolution of fever, improved well-being, and significant reduction in the frequency and productive nature of his cough. An itraconazole trough level was therapeutic at 0.5 mcg/mL (goal > 0.5 mcg/mL). Over the next two months, the patient reported resolution of all symptoms. He continues on therapeutic itraconazole with the plan of continuing lifelong suppressive therapy after one year of treatment.

## 3. Discussion

*Histoplasma* infection is a rare complication in stem cell transplant recipients with very few cases reported to date, and minimal guidance exists on how to appropriately manage this infection in this patient population [3–7].

Infection by *Histoplasma capsulatum* has been reported to occur frequently in the endemic areas of the United States in the Midwest and Central states along the Ohio and Mississippi River valleys. It is the most prevalent endemic mycosis in the United States. In the immunocompetent individual, histoplasmosis generally manifests as an asymptomatic and self-limited presentation with symptoms that are often typical of cough or nonspecific flu-like symptoms. It is reported that fewer than five percent of exposed individuals develop the symptomatic disease of progressive disseminated histoplasmosis, with common risk factors including an immunocompromised state, immunosuppressive medications, or prior solid organ transplantation [8]. In the absence of treatment, and in cases of presentation in immunocompromised individuals, severe complications can arise, including potentially fatal fibrosing mediastinitis, respiratory failure, and progressive fibrosis of lymph nodes [9]. Disseminated histoplasmosis is characterized by the progressive spread of infection to extrapulmonary sites of multiple organs and is fatal unless adequate and timely treatment is administered.

In most patients, histoplasmosis presents as a subacute pulmonary infection and is commonly mistaken as a community-acquired pneumonia or viral infection and is often only considered after there is no initial response to antibacterial therapy [10]. Symptoms typically resolve within a few weeks, but there may be persistent fatigue and asthenia that lasts for several months [11]. While most infections are self-limited, it is estimated that progressive disseminated infection occurs more commonly in immunocompromised patients.

The relationship between an individual's underlying immune function and histoplasmosis severity was elucidated in the early years of the acquired immunodeficiency syndrome (AIDS) epidemic, whereby an increased incidence in symptomatic histoplasmosis was noted [12]. Symptomatic *H. capsulatum* infection is usually accompanied by hematogenous dissemination, which in the normal host is controlled by T-cell-mediated immunity. Immunocompromised patients are unable to mount adequate immune responses and consequently become symptomatic during the period of acute dissemination [1, 9, 13].

Endemically exposed hematopoietic stem cell transplant (HSCT) recipients may be at increased risk for disseminated histoplasmosis, especially when graft-versus-host disease is managed with long-term corticosteroid therapy [1]. Reactivation of latent *Histoplasma*, rather than a newly acquired infection, is the apparent cause of disseminated histoplasmosis in immunosuppressed patients, particularly in patients from endemic areas [14]. Nevertheless, post-transplantation histoplasmosis is rare even in endemic areas. In solid organ transplant recipients, the largest single-center series reported an incidence of 1 case per 1000 person-years [15]. In a prospectively collected series of solid organ and HSCT patients diagnosed with endemic mycoses, only three of 16,200 HSCT recipients of histoplasmosis were identified (0.02%) of which only one patient underwent an allogeneic transplant [16]. Overall, there have been an estimated seven cases of histoplasmosis reported in the allogenic HSCT population, of which four patients with reported outcomes died [2, 4, 5, 7, 17, 18]. Furthermore, there have been no previously reported cases of histoplasmosis in patients where antifungal prophylaxis with agents that have clinical activity against *Histoplasma* were used as was in our reported case.

Culture of blood, respiratory samples, or bone marrow remains the accepted gold standard for diagnosis, but cultures take weeks to produce result and are often influenced by the extent of disease burden [19]. The presence of organisms in the bone marrow aspirate and biopsy is often the earliest diagnostic approach for early detection in the immunocompromised patient, especially given that the fungus may not be evident in culture for two to three weeks from the initial infection [20]. Serologic studies have not reported to be helpful in diagnosis of histoplasmosis in a prior study of immunocompromised patients, but the sensitivity of detection specifically in the bone marrow transplant population has yet to be studied [21]. Though a definitive diagnosis of histoplasmosis necessitates culture or histopathologic confirmation, a probable diagnosis can still be made in a patient with increased risk secondary to immunocompromised status, compatible clinical picture, and mycological evidence [22].

The diagnostic yield of BAL in confirming the diagnosis of a bacterial or fungal pulmonary infection is often difficult, particularly with the exposure to antibiotic or antifungal prophylaxis. Cytopathologic evaluation of the BAL fluid is relatively noninvasive and has a sensitivity of around 50% for acute pulmonary histoplasmosis; when combined with BAL *Histoplasma* antigen testing, the sensitivity increases to about 97% [23]. Antigen testing is generally accepted as the leading modality to diagnose histoplasmosis. The *Histoplasma* antigen was reformulated into an enzyme immunoassay (EIA) in 1989, and the third generation MiraVista *H. capsulatum* Galactomannan EIA became available in 2007. The MiraVista EIA *Histoplasma* antigen test was found to be the highest in patients with disseminated histoplasmosis (91.8%) and the lowest in patients with subacute histoplasmosis (30%) [24]. Detecting the urine antigen has additionally proven to be slightly more sensitive than the serum across all manifestations of histoplasmosis. In a large retrospective multicenter reviewing the incidence of histoplasmosis in a solid organ transplant population, urine *Histoplasma* antigen detection was the most sensitive diagnostic method with 93% positivity and detection of antibody the least sensitive, positive in 36% of cases [24]. The sensitivity of BAL antigen testing is generally accepted as being superior to both urine and serum (93% vs. 79% and 56%, respectively) [22]. In nondisseminated histoplasmosis, the antigen burden is lower and thus the sensitivity of antigen testing is lower, but combing the results of urine and antigen serum has demonstrated improvement in the sensitivity of detecting pulmonary histoplasmosis [25].

Molecular diagnostic methods have the advantage of higher analytic specificity and shorter turnaround times as compared to other diagnostic methodologies, especially when considering the time needed for colony growth in culture-based diagnosis [26]. There are currently no FDA-approved molecular assays for *Histoplasma* directly applicable to clinical specimens, though laboratory-based PCR assays using a variety of molecular targets have been developed. In prior studies focused in hematology-oncology patients that often have prolonged cytopenias, addition of molecular analysis testing has increased the efficacy of bronchoalveolar lavage in the diagnosis of respiratory infections [27]. In a study of 107 consecutive hospitalized patients with hematologic malignancies and bone marrow transplant recipients who underwent BAL for evaluation of pulmonary infiltrates, the addition of PCR-based *Aspergillus* DNA detection to improve the diagnostic capacity of invasive *Aspergillus* infection decreased mortality from 80 to 35.6% (*P*=0.003) [28].

Although systemic candidiasis and aspergillosis are more commonly reported as major causes of fungal infections in HSCT recipients, disseminated histoplasmosis presents a threat to the HSCT recipient from an endemic area. Furthermore, the diagnosis of histoplasmosis is especially challenging because empiric or prophylactic antifungal treatment is often administered after HSCT, and this may possibly lead to an underestimation of histoplasmosis. The significant mortality associated with post-transplant histoplasmosis and nonspecific clinical presentation further emphasizes the importance of maintaining a low threshold for considering the diagnosis of histoplasmosis. This accounts for the distinct diagnosis used between definitions of invasive fungal disease in clinical practice compared to clinical research definitions [29].

The Infectious Disease Society of America (IDSA) last published guidelines for the management of histoplasmosis in 2007 [3, 23]. In general, for patients with mild-to-moderate pulmonary histoplasmosis, treatment is not recommended. However, if patients have symptoms for greater than one month, as in the case of our patient, therapeutic itraconazole should be employed for at least one year. While there is no specific recommendation for treatment of histoplasmosis in immunocompromised patients, including recipients of allogeneic HSCT who are receiving immunosuppressive medications, the guidelines recommend that patients who are not immunocompetent should receive suppressive therapy with itraconazole if immunosuppression cannot be reversed. Furthermore, they specifically recommend prophylaxis with itraconazole in HIV patients with CD4 counts <150 cells/mm^3^ in endemic areas where the incidence is above 10 cases per 100 patient-years. Indiana, where the patient lived prior to his allogeneic stem cell transplant, is among the states that are endemic for histoplasmosis [30]. Past infection, determined by the skin test reactivity assay, occurs in 50–80% of Indiana residents [31].

Voriconazole has been reported to be successful in the treatment of a small number of patients with varying forms of *Histoplasma* infection [31–33]. The drug has demonstrated *in vitro* activity against *H. capsulatum*, but isolates that became resistant to fluconazole were noted to have elevated minimum inhibitory concentration (MIC) values to voriconazole in patients with AIDS, suggesting that resistance may develop during treatment with voriconazole [34]. IDSA recommends that voriconazole only be utilized as a second-line alternative to itraconazole for treatment of histoplasmosis. In addition, no specific prophylaxis is recommended.

Monitoring of voriconazole blood levels has been advocated as a means to ensure adequate drug exposure in treating patients with invasive mycoses. Freifeld et al. examined nine patients with disseminated histoplasmosis who failed treatment with amphotericin or itraconazole and received voriconazole as secondary therapy where serum trough levels were determined [32]. All patients were deemed to have clinically improved while receiving voriconazole 200 mg twice daily. Of the 20 samples obtained from these patients, serum voriconazole levels ranged from undetectable to 8 mcg/mL. In addition, archived *H. capsulatum* isolates from AIDS patients who had either primary or relapsed histoplasmosis were employed for MIC testing via a modified Clinical and Laboratory Standards Institute (CLIA) assay. The median MIC for primary and relapsed isolates was 0.015 mCg/mL and 0.03 mCg/mL, respectively. Despite two patients having voriconazole levels below the MIC values, no patient relapsed. The authors proposed a threshold voriconazole trough value of 0.125 mCg/mL for the treatment of histoplasmosis [32]. Notably our patient's trough level was higher at 2.4 mCg/mL.

To our knowledge, this is the first case of histoplasmosis reported in an allogeneic stem cell transplant recipient who was receiving voriconazole as antifungal prophylaxis at the appropriate serum drug levels for *Aspergillus* prophylaxis. The detection of *Histoplasma*, despite therapeutic voriconazole levels, is unique to our case. The reason for infection despite seemingly adequate coverage with voriconazole is unclear but may be related to relative resistance, though voriconazole may have contributed to his borderline positive antigen result seen on broncheoalveolar fluid antigen testing. We cannot exclude the possibility that the organism present either was intrinsically resistant or developed resistance to voriconazole due to prolonged drug exposure.

While voriconazole is not a well-validated treatment or prophylactic modality for *Histoplasma* infections, we would recommend caution in using this agent for either approach. Though molecular-based diagnostic methods have the potential to improve the diagnostic sensitivity of *Histoplasma* infections on this unique population, we recommend timely evaluation and high clinical suspicion with the current culture and serologic-based diagnostic methodology. We also recommend a lower threshold for evaluation for histoplasmosis in allogeneic transplant recipients who live in endemic regions, regardless of their antifungal prophylactic regimen.
